# Reliability analysis of the triple modular redundancy system under step-partially accelerated life tests using Lomax distribution

**DOI:** 10.1038/s41598-023-41363-3

**Published:** 2023-09-07

**Authors:** Laila A. Al-Essa, Alaa H. Abdel-Hamid, Tmader Alballa, Atef F. Hashem

**Affiliations:** 1 Department of Mathematical Sciences, College of Science, Princess Nourah bint Abdulrahman University, P.O. Box 84428, Riyadh, 11671 Saudi Arabia; 2 Department of Mathematics and Statistics, College of Science, Imam Mohammad Ibn Saud Islamic University (IMSIU), Riyadh, 11432 Saudi Arabia; 3 Mathematics and Computer Science Department, Faculty of Science, Beni-Suef University, Beni-Suef, 62511 Egypt

**Keywords:** Mathematics and computing, Statistics

## Abstract

Triple modular redundancy (TMR) is a robust technique utilized in safety-critical applications to enhance fault-tolerance and reliability. This article focuses on estimating the distribution parameters of a TMR system under step-stress partially accelerated life tests, where each component included in the system follows a Lomax distribution. The study aims to analyze the system’s reliability and mean residual lifetime based on the estimated parameters. Various estimation techniques, including maximum likelihood, percentile, least squares, and maximum product of spacings, are explored. Additionally, the optimal stress change time is determined using two criteria. An illustrative example supported by two actual data sets is presented to showcase the methodology’s application. By conducting Monte Carlo simulations, the assessment of the estimation methods’ effectiveness reveals that the maximum likelihood method outperforms the other three methods in terms of both accuracy and performance, as indicated by the numerical outcomes. This research contributes to the understanding and practical implementation of TMR systems in safety-critical industries, potentially saving lives and preventing catastrophic events.

## Introduction

### Designing the triple modular redundancy system

Within engineering systems, redundancy pertains to the replication of vital components within a system, aimed at enhancing reliability and reducing the likelihood of hazards. Systems that incorporate threefold replication of key elements are categorized as TMR systems.

A fault represents a tangible imperfection that has the potential to result in malfunctions or errors within a system. Many critical areas like electric power distribution networks, patient life support setups, transportation, nuclear reactor oversight, automobiles, aviation, and spacecraft, are required to function reliably and securely even when confronted with such imperfections, see, for example,^1–4^. Such systems demand a consistent and dependable level of service. The capability of a system to keep on performing its intended function in the presence of a fault is known as fault-tolerance^5^. Fault-tolerant systems are those that can endure more demanding environmental circumstances and external disruptions. Consequently, designers now possess a pair of choices when it comes to managing faults: either masking them or tolerating them. Errors and system failures are avoided by the technique of “fault masking”. A common example of such a technique is majority voting systems. In either scenario, redundancy (the inclusion of additional parts, units, or equipment that are not necessary for the regular running of the system) is necessary. The earliest kind of this redundancy is called TMR. At least two out of the three system output levels must be voted on in this design.

TMR is a powerful technique used in safety-critical applications to achieve high levels of fault-tolerance and reliability. By triplicating critical components and comparing their outputs, it ensures consistent operation even in the presence of faults or failures. This redundancy approach is vital in industries where the consequences of system malfunctions can be severe, potentially saving lives and preventing catastrophic events.

The majority of modules (2 out of 3) are compatible with the output within such designs. This implies that the system can tolerate a maximum of one individual module failure, which can be mitigated (masked).

**Figure 1:**
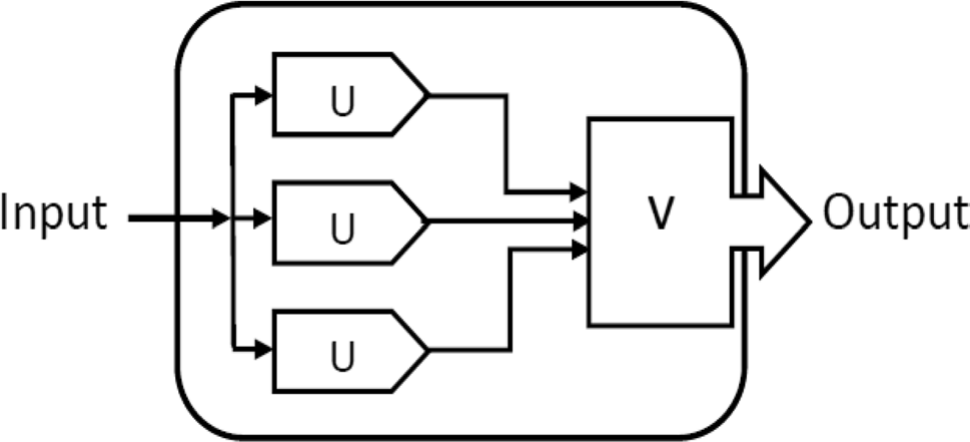
Structure of TMR system.

In Fig. 1, the unit (*U*) is replicated threefold, and these three units operate in parallel. The voting entity *V* receives the outputs from the three units. In the event that any one of the three units malfunctions, the other two units can rectify the issue. The voting component assesses the results from the three units and determines the majority decision. The TMR company can entirely conceal the unit’s failure. TMR is well suited for temporary failures because of the manner in which TMR is built, when an error occurs, the voter doesn’t delete the flawed unit.

TMR is considered a special case of a more general system called *N*-modular redundancy “NMR”. Instead of using three units, this method uses *N* units, which are typically odd numbers, allowing the system to withstand more than one unit failure^6^. For applications, features, and benefits of the TMR technique, see, for example^7,8^.

### Accelerated life tests

Ongoing enhancements in manufacturing design have led to a significant increase in the reliability of products and materials. However, acquiring data about the longevity of these enhanced reliability tools and materials through testing has become a more intricate, expensive, and time-intensive process. In situations like these, determining the hazard rates of products requires subjecting them to stress levels more severe than those encountered in the manufacturing phase. This approach, known as the “accelerated life test” (ALT), involves testing products at elevated stress levels. The hazard rates observed in ALT can then be used to approximate the expected durability of these products under typical usage conditions.

There are primarily three ALT techniques, see Ref.^9^. The first technique is referred to as the constant-stress ALT, which sustains a constant stress level throughout the entire lifespan of the tested products, see, for example^10^. The stress placed on a test product is continually increased over time in the second technique, which is known as the progressive-stress ALT, see, for example^11,12^. Several authors have explored the third technique of ALT, termed the step-stress ALT. In this method, the applied stress level is altered either after a specific number of failures have been observed or at certain predetermined time intervals.

The ALT necessitates a well-defined relation (such as the inverse power rule, Arrhenius model, log-linear model, etc.) between the stress applied to the products and their average lifespan. The parameters involved in these relations must be estimated. Often, establishing such a relation is intricate and cannot be assumed beforehand. Consequently, the extrapolation of ALT data for practical use conditions becomes unfeasible. This is where partially ALT (PALT) offers a practical solution. PALT involves determining the acceleration factor, which represents the ratio of the average lifespan under standard usage to that under accelerated conditions (as referenced in Ref.^9^). Once this factor is determined, it becomes feasible to extend the application of accelerated test data to actual usage conditions.

A step-stress PALT operates with two distinct stress levels, with the initial stress being at the standard level, and a predetermined moment when the stress is altered (as discussed in references^13–21^).

### Lomax distribution

The Lomax distribution (LD)^22^, often known as Pareto distribution of the second kind, was proposed by Lomax as a model for business failure data. It is commonly used in reliability engineering, where it is applied to model failure rates, lifetime distributions, and survival probabilities. It is also used in various fields for modeling data with heavy tails, such as insurance claims, income distribution, and extreme value analysis.

The following are, respectively, the probability density function (PDF), cumulative distribution function (CDF), reliability function (RF), and hazard rate function (HRF) of the LD, for $t>0$,

1 $$\begin{aligned} f(t)= & {} \beta \gamma \left( 1+ \beta t \right) ^{-\gamma -1}, \end{aligned}$$

2 $$\begin{aligned} F(t)= & {} 1-\left( 1+ \beta t \right) ^{-\gamma }, \end{aligned}$$

3 $$\begin{aligned} R(t)= & {} \left( 1+ \beta t \right) ^{-\gamma }, \end{aligned}$$

4 $$\begin{aligned} h(t)= & {} \frac{\beta \gamma }{1+ \beta t }, \end{aligned}$$

where $\beta >0$ and $\gamma >0$ are the scale and shape parameters of the distribution, respectively.

Several features motivate us to consider the LD as a lifetime model for the units presented in the TMR system, some of them are:

1. The CDF of the LD possesses a closed-form expression, simplifying the process of estimation.
2. The LD is characterized by a heavy right tail, indicating that extreme values occur with higher probability compared to many other distributions.
3. The parameter ($\gamma$) grants the LD more flexibility to control the tail heaviness.
4. The parameter ($\beta$) determines the spread of the distribution.
5. The LD’s HRF exhibits a decreasing trend. This characteristic could be advantageous for entities experiencing early failures. However, it might also pose a limitation, as certain scenarios might involve a bathtub-shaped hazard rate. In such instances, a Weibull distribution could be more appropriate for modeling such a situation than LD.

### *K*-out-of-*N* system

The reliability analysis of a system often concerns analyzing *K*-out-of-*N* systems. A *K*-out-of-*N* system is operational when *K* or more out of *N* units work (in other words it fails when fewer than *K* units are functional), whereas for series(parallel) systems $K=N$($K=1$).

If *R*(*t*) is the reliability of a given unit, then the *K*-out-of-*N* system’s reliability is the probability that $N-K$ or fewer units have failed by time *t* (or—at least *K* units are functional)

5 $$\begin{aligned} R_{K-\text {out}-\text {of}-N}(t)=\sum _{i=K}^{N}\left( {\begin{array}{c}N\\ i\end{array}}\right) [R(t)]^{i} [1-R(t)]^{N-i}. \end{aligned}$$

The new in this article is to analyze and estimate the reliability of the TMR system under step-stress PALTs where each component’s lifetime has a LD, through estimating the included parameters. The mean residual lifetime (MRL) as well as the HRF of the considered system are also estimated. Four different methods are considered in the estimation procedure such as the maximum likelihood (ML) estimate (MLE), percentile estimate (PE), least squares (LS) estimate (LSE), and maximum product of spacings (MPS) estimate (MPSE). Two optimal criteria are proposed to determine the ideal stress change time in the step-stress PALTs.

The article is structured into nine distinct sections, with the remaining sections outlined as follows: “Reliability of the triple modular redundancy system” presents the derivation of the reliability of the TMR system. In “Model description and mean residual lifetime”, the model is elaborated upon, accompanied by the calculation of the MRL. “Estimation methods” delves into the study of various estimation methods, including ML, percentile, LS, and MPS methods, for determining unknown parameters and parameter functions. “Common optimality criteria” introduces two criteria for the optimal timing of stress changes. The exploration of an illustrative example based on real-world data is conducted in “Real data set”. Subsequently, in “Simulation Study”, the application of Monte Carlo simulations is detailed. “Simulation results” presents selected simulation outcomes. Lastly, the article concludes with “Concluding remarks”, which offers closing observations and a glimpse into potential future research endeavors.

## Reliability of the triple modular redundancy system

Suppose that three units (components) $A_1$, $A_2$, and $A_3$ are structured in a TMR system which is set up to allow majority voting. For the system to function effectively, at least two of the three units must be in operation. Assume that *T* is a random variable (with a realization *t*) that represents the TMR system’s lifetime. If the voter’s reliability is one, the reliability of the TMR system, at time *t*, is as follows:

$$\begin{aligned} R_{\text {TMR}}(t)=R_{A_1}(t)R_{A_2}(t)(1-R_{A_3}(t))+R_{A_1}(t)R_{A_3}(t)(1-R_{A_2}(t))+R_{A_2}(t)R_{A_3}(t)(1-R_{A_1}(t))+R_{A_1}(t)R_{A_2}(t)R_{A_3}(t). \end{aligned}$$

If

$$\begin{aligned} R_{A_i}(t)=R(t),\quad i=1,2,3, \end{aligned}$$

then

6 $$\begin{aligned} R_{\text {TMR}}(t)=3R^{2}(t)-2R^{3}(t). \end{aligned}$$

It is clear that Eq. (6) coincides with Eq. (5) when $K=2$ and $N=3$. Therefore, the TMR system’s CDF and PDF are provided, respectively, by

7 $$\begin{aligned} F_{\text {TMR}}(t)= & {} 2R^{3}(t)-3R^{2}(t)+1, \end{aligned}$$

8 $$\begin{aligned} f_{\text {TMR}}(t)= & {} 6 \, f(t) \, [1-R(t)]\, R(t), \end{aligned}$$

where $f(t)=\frac{-\text {d} R(t)}{\text {d} t}$ is the PDF of lifetime of a given unit.

Using PDF (1) and RF (3) and for $(\beta , \gamma >0)$, the CDF, PDF, RF, and HRF of the TMR system are given, respectively, by

9 $$\begin{aligned} F_{\text {TMR}}(t)= & {} 2\left( 1+ \beta t \right) ^{-3\gamma }-3\left( 1+ \beta t \right) ^{-2\gamma }+1,\quad \quad t>0, \end{aligned}$$

10 $$\begin{aligned} f_{\text {TMR}}(t)= & {} 6 \beta \gamma \left( 1+ \beta t \right) ^{-2\gamma -1}\big [1-\left( 1+ \beta t \right) ^{-3\gamma }\big ], \end{aligned}$$

11 $$\begin{aligned} R_{\text {TMR}}(t)= & {} 3\left( 1+ \beta t \right) ^{-2\gamma }-2\left( 1+ \beta t \right) ^{-3\gamma }, \end{aligned}$$

12 $$\begin{aligned} h_{\text {TMR}}(t)= & {} \frac{6 \beta \gamma \left( 1+ \beta t \right) ^{-1}\big [1-\left( 1+ \beta t \right) ^{-\gamma }\big ]}{3-2\left( 1+ \beta t \right) ^{-\gamma }}. \end{aligned}$$

**Figure 2:**
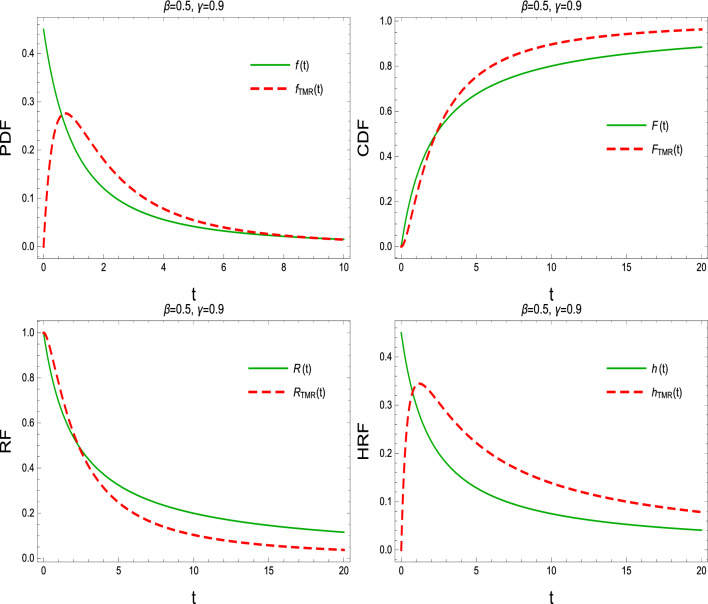
The PDF, CDF, RF, and HRF of the LD and TMR system.

The PDF, CDF, RF, and HRF of the LD and TMR system are plotted in Fig. 2 with $\beta =0.5$ and $\gamma =0.9$.

In the subsequent section, both the model’s description and its mean residual lifetime are provided.

## Model description and mean residual lifetime

This section is devoted to applying the step-stress PALT to the TMR system whose lifetime *T* is considered a random variable (with realization *t*) subjecting to CDF (9). The PDF, SF, and HRF of *T* are provided, respectively, by (10), (11), and (12). Two techniques may be used to raise the stress in the step-stress PALTs, one of them is called tampered random variable, see Ref.^23^, and the other one is called tampered failure rate (TFR), see Ref.^13^. For the TMR system under the TFR technique, as soon as a change time point $\eta$ is reached, the stress raises and remains constant. This would be done by multiplying the primary HRF $h_{\text {TMR}}(t)$ by an undefined factor $\alpha (>1)$ (which may rely on $\eta$). Let $h^{\star }(t)$ denote the total HRF in PALT. Then, the TFR model is provided by

13 $$\begin{aligned} h^\star (t)=\left\{ \begin{array}{cc} h_{\text {TMR}}(t), &{} 0<t\le \eta , \\ \alpha \, h_{\text {TMR}}(t), &{} \quad \eta<t<\infty ,\; (\alpha >1). \end{array}\right. \end{aligned}$$

For the total lifetime *T* in Model (13), the RF is provided by

14 $$\begin{aligned} R^{*}(t)=\left\{ \begin{array}{l} R_1(t)=\text {exp}\left[ -\int _0^{t}h_{\text {TMR}}(u)\text {d}u\right] ,\quad 0<t\le \eta , \\ R_2(t)=\text {exp}\left[ -\int _0^{\eta }h_{\text {TMR}}(u)\text {d}u-\int _{\eta }^{t} \alpha h_{\text {TMR}}(u)\text {d}u\right] ,\quad \eta<t<\infty . \end{array} \right. \end{aligned}$$

The function $R_2(t)$ defined in RF (14) is deduced as follows:

15 $$\begin{aligned} \begin{aligned} R_2(t)&=\text {exp}\left[ -\int _0^{\eta } \frac{f_{\text {TMR}}(u)}{R_{\text {TMR}}(u)} \text {d}u-\int _{\eta }^{t} \alpha \frac{f_{\text {TMR}}(u)}{S_{\text {TMR}}(u)} \text {d}u\right] \\&=[R_{\text {TMR}}(t)]^{\alpha }\, [R_{\text {TMR}}(\eta )]^{1-\alpha }. \end{aligned}\end{aligned}$$

By substituting RF (10) in (15), RF (15) becomes

16 $$\begin{aligned} \begin{aligned} R_2(t)&= \left[ 3\left( 1+ \beta \eta \right) ^{-2\gamma }-2\left( 1+ \beta \eta \right) ^{-3\gamma }\right] ^{1-\alpha } \left[ 3\left( 1+ \beta t \right) ^{-2\gamma }-2\left( 1+ \beta t \right) ^{-3\gamma }\right] ^{\alpha }. \end{aligned}\end{aligned}$$

Based on (10), (14) and (16), RF (14) of *T* under PALT becomes

17 $$\begin{aligned} R^{*}(t)=\left\{ \begin{array}{l} R_1(t)=3\left( 1+ \beta t \right) ^{-2\gamma }-2\left( 1+ \beta t \right) ^{-3\gamma },\quad 0<t\le \eta , \\ R_2(t)=\left[ 3\left( 1+ \beta \eta \right) ^{-2\gamma }-2\left( 1+ \beta \eta \right) ^{-3\gamma }\right] ^{1-\alpha } \left[ 3\left( 1+ \beta t \right) ^{-2\gamma }-2\left( 1+ \beta t \right) ^{-3\gamma }\right] ^{\alpha },\quad \eta<t<\infty . \end{array} \right. \end{aligned}$$

The corresponding CDF, PDF, and HRF are given, respectively, by

18 $$\begin{aligned} F^{*}(t)= & {} \left\{ \begin{array}{l} F_1(t)=1-3\left( 1+ \beta t \right) ^{-2\gamma }+2\left( 1+ \beta t \right) ^{-3\gamma },\quad 0<t\le \eta , \\ F_2(t)=1-\left( \left[ 3\left( 1+ \beta \eta \right) ^{-2\gamma }-2\left( 1+ \beta \eta \right) ^{-3\gamma }\right] ^{1-\alpha } \left[ 3\left( 1+ \beta t \right) ^{-2\gamma }-2\left( 1+ \beta t \right) ^{-3\gamma }\right] ^{\alpha }\right) ,\quad \eta<t<\infty . \end{array} \right. \end{aligned}$$

19 $$\begin{aligned} f^{*}(t)= & {} \left\{ \begin{array}{l} f_1(t)=6 \beta \gamma \left( 1+ \beta t \right) ^{-2\gamma -1}\bigg [1-\left( 1+ \beta t \right) ^{-\gamma }\bigg ],\quad 0<t\le \eta , \\ f_2(t)=6 \alpha \beta \gamma \left[ 3\left( 1+ \beta \eta \right) ^{-2\gamma }-2\left( 1+ \beta \eta \right) ^{-3\gamma }\right] ^{1-\alpha } \left[ 3\left( 1+ \beta t \right) ^{-2\gamma }-2\left( 1+ \beta t \right) ^{-3\gamma }\right] ^{\alpha -1}\,\\ \quad \quad \quad \times \left( 1+ \beta t \right) ^{-2\gamma -1}\big [1-\left( 1+ \beta t \right) ^{-\gamma }\big ],\quad \eta<t<\infty . \end{array} \right. \end{aligned}$$

20 $$\begin{aligned} h^{*}(t)= & {} \left\{ \begin{array}{l} h_1(t)=\displaystyle \frac{6 \beta \gamma \left( 1+ \beta t \right) ^{-1}\big [1-\left( 1+ \beta t \right) ^{-\gamma }\big ]}{3-2\left( 1+ \beta t \right) ^{-\gamma }},\quad 0<t\le \eta , \\ h_2(t)=\displaystyle \frac{6 \alpha \beta \gamma \left( 1+ \beta t \right) ^{-1}\big [1-\left( 1+ \beta t \right) ^{-\gamma }\big ]}{3-2\left( 1+ \beta t \right) ^{-\gamma }},\quad \eta<t<\infty . \end{array} \right. \end{aligned}$$

### Mean residual lifetime

For the purposes of studying survival analysis, the MRL is crucial. Assuming the system has lived up until time $t_0$, the MRL is defined as the expected residual lifetime as follows:

$$\begin{aligned} \begin{aligned} m(t_{0})&=\text {E}[(T-t_{0})\mid T>t_{0}]\\&=\frac{1}{R^{*}(t_{0})} \int _{t_{0}}^{\infty } R^{*}(t){\text{ d }}t. \end{aligned} \end{aligned}$$

21 $$\begin{aligned} \left. \begin{aligned} \text {If}\quad t_{0}\le \eta&\\ m(t_{0})&=\frac{1}{R_{1}(t_{0})} \left[ \int _{t_{0}}^{\eta } R_{1}(t){\text{ d }}t + \int _{\eta }^{\infty } R_{2}(t){\text{ d }}t \right] \\&=\frac{1}{3\left( 1+ \beta t_{0} \right) ^{-2\gamma }-2\left( 1+ \beta t_{0} \right) ^{-3\gamma }} \Bigg (\int _{t_{0}}^{\eta } \left[ 3\left( 1+ \beta t \right) ^{-2\gamma }-2\left( 1+ \beta t \right) ^{-3\gamma } \right] {\text{ d }}t \\&\quad \quad + \left[ 3\left( 1+ \beta \eta \right) ^{-2\gamma }-2\left( 1+ \beta \eta \right) ^{-3\gamma }\right] ^{1-\alpha } \int _{\eta }^{\infty } \left[ 3\left( 1+ \beta t \right) ^{-2\gamma }-2\left( 1+ \beta t \right) ^{-3\gamma }\right] ^{\alpha } {\text{ d }}t \Bigg ),\\ \text {If}\quad t_{0}> \eta&\\ m(t_{0})&=\frac{1}{R_{2}(t_{0})} \int _{t_{0}}^{\infty } R_{2}(t){\text{ d }}t\\&=\frac{1}{ \left[ 3\left( 1+ \beta t_{0} \right) ^{-2\gamma }-2\left( 1+ \beta t_{0} \right) ^{-3\gamma }\right] ^{\alpha }} \int _{t_{0}}^{\infty } \left[ 3\left( 1+ \beta t \right) ^{-2\gamma }-2\left( 1+ \beta t \right) ^{-3\gamma }\right] ^{\alpha }{\text{ d }}t. \end{aligned}\right\} \end{aligned}$$

## Estimation methods

In this section, the parameters $\alpha ,\beta ,$ and $\gamma$ of the TMR system under step-stress PALT with CDF (18) and PDF (19) are estimated using four estimation methods which are the ML, percentile, least squares, and MPS. RF (17), HRF (20), and MRL function (21) are also estimated using the invariance property (IP).

It is common knowledge that the method of ML estimation is enjoyed with the IP. This property does not apply well to the estimation of a parametric function when the approach of parameter estimation is switched from ML to any other conventional method. The IP of the MPS estimation was studied by Anatolyev and Kosenok^24^, who came to the conclusion that it shares the same property as ML estimation. However, different authors have attempted to adopt this IP for other best estimators of the parameters to estimate a function of the parameters in various contexts, see, for example, Srinivasa Rao and Kantam^25^.

### Maximum likelihood estimation

Let’s assume that ${{\textbf {t}}}=(t_{(1)}<t_{(2)}<\dots<t_{(n_1)}\le \eta<t_{(n_1+1)}<\dots <t_{(n)})$ is an ordered observed random sample taken from a population with PDF (19), where $n_1(n_2=n-n_1)$ is a random number denoting the number of failures under normal(accelerated) stress condition. Consequently, the likelihood function can be expressed as follows:

22 $$\begin{aligned} \begin{aligned} \mathbb {L}(\alpha ,\beta ,\gamma ;{{\textbf {t}}})&\varpropto \left[ \prod ^{n_{1}}_{j=1} f_{1}(t_{(j)})\right] \left[ \prod ^{n}_{j=n_{1}+1} f_{2}(t_{(j)})\right] . \end{aligned} \end{aligned}$$

In general, it is simpler to determine the MLEs of the underlying parameters by maximizing the natural logarithm of the likelihood function than the likelihood function itself. Thus, using PDF (19), if $\mathcal {L}=\ln \mathbb {L}(\alpha ,\beta ,\gamma ;{{\textbf {t}}})$, then

23 $$\begin{aligned} \begin{aligned} \mathcal {L}&\varpropto n \ln [6\beta \gamma ] +n_{2} \ln [\alpha ] - (2\gamma +1) \sum ^{n}_{j=1} \ln \left[ 1+\beta t_{(j)}\right] + \sum ^{n}_{j=1} \ln \left[ 1-(1+\beta t_{(j)})^{-\gamma }\right] \\&\quad +n_{2} (1-\alpha )\ln \left[ 3(1+\beta \eta )^{-2\gamma }-2(1+\beta \eta )^{-3\gamma }\right] \\&\quad + (\alpha -1) \sum ^{n}_{j=n_{1}+1} \ln \left[ 3(1+\beta t_{(j)})^{-2\gamma }-2(1+\beta t_{(j)})^{-3\gamma }\right] . \end{aligned} \end{aligned}$$

Based on (23), by solving the likelihood equations with respect to $\alpha ,\beta$, and $\gamma$ after equating them to zero, the MLEs $(\hat{\alpha },\hat{\beta },\hat{\gamma })$ of $(\alpha ,\beta ,\gamma )$ can be obtained. This procedure can be done as follows:

24 $$\begin{aligned}{} & {} \frac{\partial \mathcal {L}}{\partial \alpha }=0= \frac{n_{2}}{\alpha }-n_{2} \ln \left[ 3(1+\beta \eta )^{-2\gamma }-2(1+\beta \eta )^{-3\gamma }\right] + \sum ^{n}_{j=n_{1}+1} \ln \left[ 3(1+\beta t_{(j)})^{-2\gamma }-2(1+\beta t_{(j)})^{-3\gamma }\right] , \end{aligned}$$

25 $$\begin{aligned}{} & {} \frac{\partial \mathcal {L}}{\partial \beta }=0= \frac{n}{\beta }+ 6 n_{2} \eta \gamma (1-\alpha ) \frac{(1+\beta \eta )^{-\gamma -1} -(1+\beta \eta )^{-1} }{3-2(1+\beta \eta )^{-\gamma }} - \sum ^{n}_{j=1}\frac{t_{(j)}}{1+\beta t_{(j)}} \left( 2\gamma +1 -\frac{\gamma }{(1+\beta t_{(j)})^{\gamma }-1}\right) \nonumber \\{} & {} \quad + 6\gamma (\alpha -1) \sum ^{n}_{j=n_{1}+1} t_{(j)} \frac{(1+\beta t_{(j)})^{-\gamma -1} -(1+\beta t_{(j)})^{-1} }{3-2(1+\beta t_{(j)})^{-\gamma }}, \end{aligned}$$

26 $$\begin{aligned}{} & {} \frac{\partial \mathcal {L}}{\partial \gamma }=0= \frac{n}{\gamma } + 6 n_{2} (1-\alpha ) \ln [1+\beta \eta ] \left( \frac{(1+\beta \eta )^{-\gamma }-1}{3-2 (1+\beta \eta )^{-\gamma } }\right) -\sum ^{n}_{j=1} \ln [1+\beta t_{(j)}] \left( 2-\frac{1}{(1+\beta t_{(j)})^{\gamma }-1} \right) \nonumber \\{} & {} \quad + 6 (\alpha -1) \sum ^{n}_{j=n_{1}+1} \ln [1+\beta t_{(j)}] \left( \frac{(1+\beta t_{(j)})^{-\gamma }-1}{3-2 (1+\beta t_{(j)})^{-\gamma } } \right) . \end{aligned}$$

From (24), the following equation can be used to calculate $\hat{\alpha }$ as a function of $\beta$ and $\gamma$,

27 $$\begin{aligned} \begin{aligned} \hat{\alpha }(\beta ,\gamma )= n_{2} {\Bigg /} \left( n_{2} \ln \left[ 3(1+\beta \eta )^{-2\gamma }-2(1+\beta \eta )^{-3\gamma }\right] - \sum ^{n}_{j=n_{1}+1} \ln \left[ 3(1+\beta t_{(j)})^{-2\gamma }-2(1+\beta t_{(j)})^{-3\gamma }\right] \right) . \end{aligned} \end{aligned}$$

By substituting $\hat{\alpha }(\beta ,\gamma )$ in (25) and (26), the MLEs of $\beta$ and $\gamma$ can be produced by solving the likelihood equations $\displaystyle \frac{\partial \mathcal {L}}{\partial \beta }=0$ and $\displaystyle \frac{\partial \mathcal {L}}{\partial \gamma }=0$, with regard to $\beta$ and $\gamma$ by utilizing any numerical iteration method.

The MLEs of RF, HRF, and MRL function at time $t_{0}$, denoted by $\hat{R}(t_{0})$, $\hat{h}(t_{0})$ and $\hat{m}(t_{0})$, are obtained by substituting $(\hat{\alpha },\hat{\beta },\hat{\gamma })$ in Eqs. (17), (20), and (21), respectively.

#### Remark 4.1

It can be noticed that:

1. The MLEs of $\alpha$, $\beta$, and $\gamma$ don’t exist if there are no failures observable under normal stress circumstances ($n_{1}=0$ and $n_2=n$).
2. The MLE of $\alpha$ does not exist if there are no failures observable under accelerated stress circumstances ($n_1=n$ and $n_{2}=0$).
3. The MLEs of $\alpha$, $\beta$, and $\gamma$ exist if there are at least two failures observable during the test one of them is under normal stress circumstances ($n_{1}>0$ and $n_{2}>0$).

The initial two aspects outlined in Remark 4.1 could potentially serve as limitations when applying the model under certain specific data.

In light of the widespread asymptotic normality theory for MLEs, each of the following standard quantities $(\hat{\alpha } -\alpha )/\sqrt{\text {Var}(\hat{\alpha } )}$, $(\hat{\beta } -\beta )/\sqrt{\text {Var}(\hat{\beta } )}$, and $(\hat{\gamma } -\gamma )/\sqrt{\text {Var}(\hat{\gamma } )}$ can subject to a normal distribution with mean 0 and variance 1, where $\text {Var}(\hat{\alpha })$, $\text {Var}(\hat{\beta })$, and $\text {Var}(\hat{\gamma })$ stand for the variances, of the MLEs, which correspond to the main diagonal of the inverse of observed Fisher information matrix (FIM) given by

28 $$\begin{aligned} \begin{aligned} \mathbb {V}= \Psi ^{-1} =&\left( \begin{array}{ccc} \text {Var}(\hat{\alpha }) &{}\quad \text {Cov}(\hat{\alpha }, \hat{\beta }) &{}\quad \text {Cov}(\hat{\alpha }, \hat{\gamma })\\ \text {Cov}(\hat{\alpha }, \hat{\beta })&{}\quad \text {Var}(\hat{\beta }) &{}\quad \text {Cov}(\hat{\beta }, \hat{\gamma })\\ \text {Cov}(\hat{\alpha }, \hat{\gamma })&{}\quad \text {Cov}(\hat{\beta }, \hat{\gamma }) &{}\quad \text {Var}(\hat{\gamma }) \end{array}\right) , \end{aligned} \end{aligned}$$

where

29 $$\begin{aligned} \begin{aligned} \Psi =-&\left( \begin{array}{ccc} \displaystyle \frac{\partial ^2\hat{\pounds }}{\partial \alpha ^{2}} &{}\quad \displaystyle \frac{\partial ^2\hat{\pounds }}{\partial \alpha \partial \beta } &{}\quad \displaystyle \frac{\partial ^2\hat{\pounds }}{\partial \alpha \partial \gamma } \\ \displaystyle \frac{\partial ^2\hat{\pounds }}{\partial \beta \partial \alpha }&{}\quad \displaystyle \frac{\partial ^2\hat{\pounds }}{ \partial \beta ^{2}} &{}\quad \displaystyle \frac{\partial ^2\hat{\pounds }}{\partial \beta \partial \gamma } \\ \displaystyle \frac{\partial ^2\hat{\pounds }}{\partial \gamma \partial \alpha } &{}\quad \displaystyle \frac{\partial ^2\hat{\pounds }}{\partial \gamma \partial \beta } &{}\quad \displaystyle \frac{\partial ^2\hat{\pounds }}{\partial \gamma ^{2}} \end{array}\right) , \end{aligned} \end{aligned}$$

where the caret $\hat{}$ means that the derivative is calculated at the MLEs of the parameters. Matrix (28) is useful in determining confidence intervals (CIs) for the unknown parameters. It is simple to determine the elements of Matrix (29).

Consider $\mu _1=\alpha$, $\mu _2=\beta$ and $\mu _3=\gamma$. Thus, a normal approximation CI (NACI) for $\mu _i$, with confidence coefficient $100(1-\rho )\%$, could be given as follows:

$$\begin{aligned} \left( \text {max}\{0, \hat{\mu _i} - z_{\rho /2}\sqrt{\text {Var}(\hat{\mu _i} )}\}, \hat{\mu _i} + z_{\rho /2}\sqrt{\text {Var}(\hat{\mu _i} )}\right) , \end{aligned}$$

where, for $i=1,2,3$, $\hat{\mu _i}$ is the MLE of $\mu _i$ and $P(Z>z_{\rho /2})=\rho /2$, $Z\sim N(0,1)$.

A negative value for the positive parameter often appears in the lower bound of NACI. Hence, Meeker and Escobar^26^ recommended adopting a log transformation CI (LTCI) for this parameter. Therefore, for $i=1,2,3$, $\displaystyle \frac{\ln \hat{\mu _i} -\ln \mu _i}{\sqrt{\text {Var}(\ln \hat{\mu _i} )}}\sim N(0,1)$, according to the normal approximation of log-transformed MLE, where $\text {Var}(\ln \hat{\mu _i} )=\displaystyle \frac{\text {Var}(\hat{\mu _i} )}{\hat{\mu _i}^{2}}$.

Thus, a LTCI for $\mu _i$, with confidence coefficient $100(1-\rho )\%$, could be given as follows:

$$\begin{aligned} \left( {\hat{\mu _i}}\,{\exp \left[ -{z_{\rho /2}\displaystyle \frac{\sqrt{\text {Var}(\hat{\mu _i} )}}{\hat{\mu _i}}}\right] }, {\hat{\mu _i}}\,{\exp \left[ {z_{\rho /2}\displaystyle \frac{\sqrt{\text {Var}(\hat{\mu _i} )}}{\hat{\mu _i}}}\right] }\right) . \end{aligned}$$

The second estimation method, percentile estimation, is discussed in the following subsection.

### Percentile estimation

Kao^27^ proposed a percentile approach to compute estimates for unknown parameters. This method proves suitable for estimating an unknown parameter when collecting data using a closed-form CDF. It involves adjusting a linear relationship between the percentile values obtained from the sample and the corresponding theoretical points derived from the CDF.

Suppose that, for $i=1,\dots , n$, $t_{(i)}$ is a realization of the $i\hbox {th}$ order statistic $T_{(i)}$ in a random sample of size *n* taken from a population with PDF (19). If $\Omega _{i}$ is an estimate of $F(t_{(i)})$, then minimization of the next equation with regard to $\alpha , \beta$ and $\gamma$, will yield the PEs $\check{\alpha }$, $\check{\beta }$, and $\check{\gamma }$.

30 $$\begin{aligned} \begin{aligned} \varvec{\Upsilon }(\alpha ,\beta ,\gamma ;{{\textbf {t}}})=&\sum _{i=1}^{n_{1}} \Bigg (1+ 2(1+\beta t_{(i)})^{-3\gamma } -3(1+\beta t_{(i)})^{-2\gamma }- \Omega _{i} \Bigg )^{2} + \sum _{i=n_{1}+1}^{n}\Bigg ( 2(1+\beta t_{(i)})^{-3\gamma } -3(1+\beta t_{(i)})^{-2\gamma }\\&+\bigg [(1-\Omega _{i}) \Big ( 3(1+\beta \eta )^{-2\gamma }-2(1+\beta \eta )^{-3\gamma } \Big )^{\alpha -1} \bigg ]^{1/\alpha } \Bigg )^{2}, \end{aligned} \end{aligned}$$

where $$\Omega _{i}=\displaystyle \frac{2\,i-1}{2\,n}$$ and $${{\textbf {t}}}=(t_{(1)},t_{(2)},\dots , t_{(n)}$$).

Some other approximations for $\Omega _{i}$ were proposed in statistical literature, see, for example,^28^ and^29^. Minimization of Eq. (30) can be achieved by solving $\displaystyle {\frac{\partial \varvec{\Upsilon }}{\partial \alpha }=0}$, $\displaystyle {\frac{\partial \varvec{\Upsilon }}{\partial \beta }=0}$, and $\displaystyle {\frac{\partial \varvec{\Upsilon }}{\partial \gamma }=0}$ with regard to $\alpha$, $\beta$, and $\gamma$.

The PEs of RF, HRF, and MR lifetime function at time $t_{0}$, denoted by $\check{R}(t_{0})$, $\check{h}(t_{0})$, and $\check{m}(t_{0})$, are obtained by substituting $(\check{\alpha },\check{\beta },\check{\gamma })$ in Eqs. (17), (20), and (21), respectively.

### Least squares estimation method

Swain et al.^30^ determined the LSEs of the parameters included in Beta distribution. Suppose that, for $i=1,\dots , n$, $t_{(i)}$ is a realization of the *i*th order statistic $T_{(i)}$ in a random sample of size *n* taken from a population with PDF (19). The expectation of the empirical CDF $F(t_{(i)})$ is as follows:

$$\begin{aligned} \begin{aligned} \text {E}[ F(T_{(i)})]&=\displaystyle \frac{i}{n+1}. \end{aligned} \end{aligned}$$

Minimization of the next equation with regard to $\alpha , \beta$ and $\gamma$, will yield the LSEs $\ddot{\alpha }$, $\ddot{\beta }$, and $\ddot{\gamma }$,

31 $$\begin{aligned} \begin{aligned} \mathbb {S^{*}}(\alpha , \beta ,\gamma ;{{\textbf {t}}})&=\sum _{i=1}^{n_{1}} \left( F_{1}(t_{(i)})-\displaystyle \frac{i}{n+1}\right) ^{2} +\sum _{i=n_{1}+1}^{n} \left( F_{2}(t_{(i)})-\displaystyle \frac{i}{n+1}\right) ^{2}\\&= \sum _{i=1}^{n_{1}} \Bigg (1-3\left( 1+ \beta t_{(i)} \right) ^{-2\gamma }+2\left( 1+ \beta t_{(i)} \right) ^{-3\gamma }-\displaystyle \frac{i}{n+1}\Bigg )^{2}\\&\quad + \sum _{i=n_{1}+1}^{n}\Bigg ( 1-\left( \left[ 3\left( 1+ \beta \eta \right) ^{-2\gamma }-2\left( 1+ \beta \eta \right) ^{-3\gamma }\right] ^{1-\alpha } \left[ 3\left( 1+ \beta t_{(i)} \right) ^{-2\gamma }-2\left( 1+ \beta t_{(i)} \right) ^{-3\gamma }\right] ^{\alpha }\right) -\displaystyle \frac{i}{n+1} \Bigg )^{2}, \end{aligned} \end{aligned}$$

where $${{\textbf {t}}}=(t_{(1)},t_{(2)},\dots , t_{(n)})$$.

Minimization of Eq. (31) can be achieved by solving $\displaystyle {\frac{\partial \mathbb {S^{*}}}{\partial \alpha }=0}$, $\displaystyle {\frac{\partial \mathbb {S^{*}}}{\partial \beta }=0}$ and $\displaystyle {\frac{\partial \mathbb {S^{*}}}{\partial \gamma }=0}$ with regard to $\alpha$, $\beta$, and $\gamma$. This can be done numerically by using Newton–Raphson technique, say.

By substituting $(\ddot{\alpha },\ddot{\beta },\ddot{\gamma })$ in Eqs. (17), (20), and (21), respectively, the LSEs of RF, HRF, and MR lifetime function at time $t_{0}$, denoted by $\ddot{R}(t_{0})$, $\ddot{h}(t_{0})$, and $\ddot{m}(t_{0})$, are obtained.

### Maximum product of spacings estimation

The MPS technique, suggested in Ref.^31^, is an alternative to the ML method for estimating the unknown parameters in continuous distributions. For CDF (18), define

32 $$\begin{aligned} \begin{aligned} D_{i}(\alpha ,\beta ,\gamma )= F^{*}(t_{(i)})-F^{*}(t_{(i-1)}), \quad i=1,\dots , n+1, \end{aligned} \end{aligned}$$

where $$\sum _{i=1}^{n+1}D_{i}(\alpha ,\beta ,\gamma )=1$$, $$F^{*}(t_{0})=0$$, and $$F^{*}(t_{(n+1)})=1$$.

Maximization of the geometric mean of the uniform spacings, given in the next equation, will yield the MPSEs $(\bar{\alpha },\bar{\beta },\bar{\gamma })$ of $(\alpha ,\beta ,\gamma ;{{\textbf {t}}})$,

33 $$\begin{aligned} \begin{aligned} G(\alpha ,\beta ,\gamma )&=\left[ \prod _{i=1}^{n+1} D_{i}(\alpha ,\beta ,\gamma )\right] ^{1/n+1}\\&=\left[ \left( \prod _{i=1}^{n_{1}} \left[ F_{1}(t_{(i)})-F_{1}(t_{(i-1)})\right] \right) \Bigg ( F_{2}(t_{(n_1+1)})-F_{1}(t_{(n_1)})\Bigg ) \left( \prod _{i=n_{1}+2}^{n+1}\left[ F_{2}(t_{(i)})-F_{2}(t_{(i-1)})\right] \right) \right] ^{1/n+1}, \end{aligned} \end{aligned}$$

or equivalently, by maximization of the following function, $M(\alpha ,\beta ,\gamma ;{{\textbf {t}}})=\ln [G(\alpha ,\beta ,\gamma ;{{\textbf {t}}})]$,

34 $$\begin{aligned} \begin{aligned} M(\alpha ,\beta ,\gamma ;;{{\textbf {t}}})=\frac{1}{n+1}\left[ \sum _{i=1}^{n_{1}} \ln \left[ F_{1}(t_{(i)})-F_{1}(t_{(i-1)})\right] +\ln [ F_{2}(t_{(n_1+1)})-F_{1}(t_{(n_1)})] +\sum _{i=n_{1}+2}^{n+1} \ln \left[ F_{2}(t_{(i)})-F_{2}(t_{(i-1)})\right] \right] . \end{aligned} \end{aligned}$$

Maximization of (34) can be achieved by solving the equations $\displaystyle { \frac{\partial M}{\partial \alpha }} =0$, $\displaystyle { \frac{\partial M}{\partial \beta }} =0$, and $\displaystyle { \frac{\partial M}{\partial \gamma }} =0$ with regard to $\alpha , \beta$, and $\gamma$. This can be done numerically by using Newton-Raphson technique, say.

The MPSEs of RF, HRF, and MR lifetime function at time $t_{0}$, denoted by $\bar{R}(t_{0})$, $\bar{h}(t_{0})$, and $\bar{m}(t_{0})$, are obtained by substituting $(\bar{\alpha },\bar{\beta },\bar{\gamma })$ in Eqs. (17), (20), and (21), respectively.

## Common optimality criteria

In this section, the focus shifts to the matter of ascertaining the most suitable time point $\eta$ for elevating the stress level. Two criteria, A-optimality and D-optimality, come into play as methods for identifying the optimal $\eta$ value, see Refs.^32–36^.

1. A-optimality criterion
  The primary goal of this criterion is to maximize the FIM’s trace, which provides a measure of total information based on marginal knowledge about the parameters. It is preferred to use this criterion when the estimates are at most moderately correlated.
  Maximization of the trace of FIM, (29), determined at the MLEs $\hat{\alpha }$, $\hat{\beta }$, and $\hat{\gamma }$, will yield the optimal value $\eta ^{*}_{A}$ of $\eta$, i.e.
  $$\begin{aligned} \text {Maximize}\{ tr(\Psi )\}. \end{aligned}$$
2. D-optimality criterion
  The primary goal of this criterion is to maximize the FIM’s determinant which offers a comprehensive assessment of variability by taking into consideration the correlation between the estimates. When the estimates are strongly correlated, this criterion is preferred. Consequently, maximization(minimization) of the determinant of FIM(inverse of FIM) (28, 29), determined at the MLEs $\hat{\alpha }$, $\hat{\beta }$, and $\hat{\gamma }$, will yield the optimal value $\eta ^{*}_{D}$ of $\eta$, i.e.
  $$\begin{aligned} \text {Maximize}\{ |\Psi |\} =\text {Minimize}\{ |\Psi ^{-1}|\}, \end{aligned}$$
  where $${{\textbf {t}}}=(t_{(1)},t_{(2)},\dots , t_{(n)})$$.

Maximization of the $tr(\Psi )$ [$|\Psi |$] can be achieved by solving the equation $\displaystyle \frac{\partial tr(\Psi )}{\partial \eta }=0$ $\left[ \displaystyle \frac{\partial |\Psi |}{\partial \eta }=0\right]$, with regard to $\eta$. The solution cannot be represented in a closed-form. An iterative method must be used to reach a numerical solution.

## Real data set

Two real sets of data are taken into consideration in this section to clarify the inference techniques carried out in “Estimation methods” and the optimality criteria stated in “Common optimality criteria”.

- The first real set of data was proposed in^37^. The reliability properties of solar lighting equipment were evaluated using a step-stress test of two levels. As soon as the time point $\eta = 5$ is reached, the temperature is raised from 293 to 353 K when some devices are placed through a life test at their regular working temperature (in hundred hours). The data set is listed in Table 1 where one can see that 15 devices failed under the first stress level, while 16 devices failed under the second stress level.
- The second real set of data was proposed in Ref.^38^. A prototype small/micro unmanned aerial vehicle’s (SUAV) reliability characteristics for civil applications, such as disaster management, fire monitoring, and forest aerial surveys, were evaluated using a step-stress test of two levels. The SUAV is an aircraft that is operated remotely by a ground operator while being autonomously controlled by an automatic system. Dual independent propulsion systems were included with the SUAV. In the event that both systems malfunction, the SUAV loses its flying height prior to finishing its mission. The SUAV is not affected by the failure of either propulsion system, despite the failure time being captured digitally. The stress factor whose level was altered during the test, in this instance, is the wind speed. $\eta =15$ is the stress change time. Table 1, in the last rows, lists the data set.

**Figure 3:**
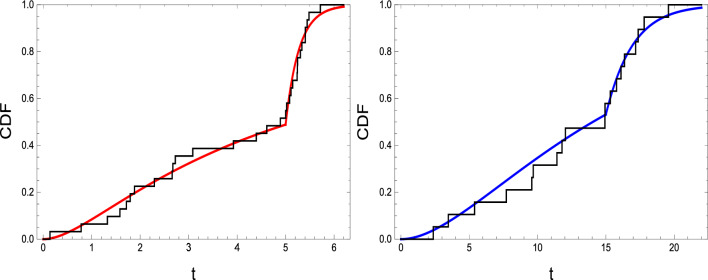
CDF (18) together with empirical CDF for the data given in Table 1.

Now, it is reasonable to inquire whether CDF (18) matches the two real sets of data that are shown in Table 1. A test statistic, related to Kolmogorov–Smirnov (KS), and its associated *p*-value are employed. It is evident from the results shown in Table 2 that every *p*-value exceeds 0.05 indicating that the model with CDF (18) matches the two real sets of data presented well. Drawing the empirical CDF alongside CDF (18) for each real set of data further illustrates this point, see Figure 3.

Based on the data displayed in Tables 1, 3 displays the MLEs, MPSEs, PEs, LSEs, and WLSEs of the parameters $\alpha$, $\beta$, and $\gamma$. Also, it displays the estimates of RF and HRF as well as the MRL. Furthermore, the NACIs and LTCIs of the model parameters in addition to the optimal value of increasing stress level ($\eta$) under two optimality criteria, $\eta ^{*}_{A}$ and $\eta ^{*}_{D}$, are calculated and presented in Table 4.

**Table 1:** The two real sets of data.

The first real set of data	
Normal stress (before $\eta =5.0$)	0.140, 0.783, 1.324, 1.582, 1.716, 1.794, 1.883, 2.293, 2.660, 2.674, 2.725, 3.085, 3.924, 4.396, 4.612, 4.892
Accelerated stress (after $\eta =5.0$)	5.002, 5.022, 5.082, 5.112, 5.147, 5.238, 5.244, 5.247, 5.305, 5.337, 5.407, 5.408, 5.445, 5.483, 5.717
The second real set of data	
Normal stress (before $\eta =15.0$)	2.365, 3.467, 5.386, 7.714, 9.578, 9.683, 11.416, 11.789, 12.039, 14.928, 14.938
Accelerated stress (after $\eta =15.0$)	15.325, 15.781, 16.105, 16.362, 17.178, 17.366, 17.803, 19.578

**Table 2:** MLEs of the model parameters, KS statistic value, and *p*-value for the two real data sets.

	MLE			KS	*p*-value
	$\hat{\alpha }$	$\hat{\beta }$	$\hat{\gamma }$		
First data set	27.7471	0.3196250	0.710198	0.0719605	0.9971
Second data set	7.34072	0.0126339	0.245030	0.0663073	0.9999

**Table 3:** MLEs, MPSEs, PEs, and LSEs of $(\alpha ,\beta , \gamma )$, RF, HRF, as well as the MRL based on the real data given in Table 1.

First data set						
	${\hat{\alpha }}$	${\hat{\beta }}$	${\hat{\gamma }}$	${\hat{S}}(5.5)$	${\hat{h}}(5.5)$	${\hat{m}}(5.5)$
MLE	27.7471	0.319625	0.710198	0.08713	3.47124	0.29476
MPSE	20.766	0.226376	0.951661	0.09971	3.09517	0.32977
PE	27.4381	0.214792	0.960972	0.06505	3.99833	0.25383
LSE	20.9017	0.275649	0.805379	0.11750	2.83216	0.36232

Second data set						
	${\hat{\alpha }}$	${\hat{\beta }}$	${\hat{\gamma }}$	${\hat{S}}(17)$	${\hat{h}}(17)$	${\hat{m}}(17)$
MLE	7.34072	0.0126339	4.24503	0.168441	0.514581	1.93075
MPSE	6.1515	0.0064666	7.9037	0.194078	0.449505	2.18543
PE	8.95116	0.00713547	6.816	0.151165	0.605719	1.62737
LSE	8.36036	0.0254261	2.1372	0.187362	0.492314	2.04789

**Table 4:** NACIs, and LTCIs of $(\alpha ,\beta , \gamma )$ and optimal stress change values, $\eta ^{*}_{D}$ and $\eta ^{*}_{A}$ based on the real data given in Table 1.

	NACI($\alpha$)	LTCI($\alpha$)	
	NACI($\beta$)	LTCI($\beta$)	$\eta ^{*}_{A}$
	NACI($\gamma$)	LTCI($\gamma$)	$\eta ^{*}_{D}$
First data set	(6.0036, 49.4906)	(12.6733, 60.7498)	1.2605
	(0.0000, 0.7788)	(0.0760, 1.3443)	4.8744
	(0.0000, 1.4212)	(0.2610, 1.9327)	
Second data set	(0.7547, 13.9267)	(2.9929, 18.0046)	10.3639
	(0.0000, 0.0480)	(0.0008, 0.2083)	14.1366
	(0.0000, 15.2864)	(0.3150, 57.2124)	

## Simulation study

One of the valuable techniques for evaluating and contrasting the efficiency of estimation methods is the utilization of Monte Carlo simulation. Within this section, we engage in numerical exploration through Monte Carlo simulation to appraise the effectiveness and proficiency of the estimation approaches and optimality criteria discussed in the previous sections. The ensuing sequence of actions is outlined below:

1. Provide values for the parameters $(\alpha , \beta , \gamma )$, the sample size *n*, and $\eta$ (stress change time).
2. From the uniform distribution with support (0, 1), generate a sample of *n* observations, $(V_1,V_2,\dots , V_n)$.
3. For $i=1,\dots ,n$, generate two observations $t_{1,i}$ and $t_{2,i}$ from CDFs $F_1(t)$ and $F_2(t)$, respectively, given by (18). Therefore, the observations $(t_1, t_2, \dots , t_n)$ under PALT from CDF (18) can be obtained as follows:
  $$\begin{aligned} t_{i}= & {} t_{1,i}^{\epsilon _i} .\, t_{2,i}^{1-\epsilon _i}, \\ \epsilon _{i}= & {} \left\{ \begin{array}{cc} 1, &{}\quad \quad t_{1,i}\le \eta , \\ 0, &{}\quad \quad t_{1,i}> \eta , \end{array} \right. \end{aligned}$$
  where,
  $$\begin{aligned}{} & {} t_{1, i}=\text {Solution} \left\{ 1+ 2(1+\beta t_{i})^{-3\gamma } -3(1+\beta t_{i})^{-2\gamma }- V_{i}=0\right\} ,\\{} & {} t_{2, i}=\text {Solution} \left\{ 2(1+\beta t_{i})^{-3\gamma } -3(1+\beta t_{i})^{-2\gamma }+\bigg [(1-V_{i}) \Big ( 3(1+\beta \eta )^{-2\gamma }-2(1+\beta \eta )^{-3\gamma } \Big )^{\alpha -1} \bigg ]^{1/\alpha } =0\right\} . \end{aligned}$$
4. Find the value of $n_{1}$, the number of observations that occur under normal stress conditions, as follows:
  $$\begin{aligned} t_{n_{1}:n}\le \eta < t_{n_{1}+1:n}. \end{aligned}$$
5. Calculate the MLEs, MPSEs, PEs, LSEs, NACIs, and LTCIs of $(\alpha ,\beta , \gamma )$.
6. Calculate the MLEs, MPSEs, PEs, and LSEs of the RF and HRF as well as the MRL (at time $t=5.0$).
7. Calculate the values of $\eta ^{*}_{D}$ and $\eta ^{*}_{A}$ (the optimal values at which the stress can be raised).
8. Replicate the above steps $\mathbb {M}$(= 5000) times.
9. The average of estimates with their mean squared error (MSE), and relative absolute bias (RAB) of $\hat{\varrho }$ over $\mathbb {M}$ samples are calculated according to the following:
  $$\begin{aligned} \overline{\widehat{\varrho }}=\frac{1}{\mathbb {M}}\sum _{i=1}^{\mathbb {M}}\hat{\varrho }_{i},\quad \quad \quad \text {RAB}(\hat{\varrho })=\frac{1}{\mathbb {M}}\sum _{i=1}^{\mathbb {M}}\frac{|\hat{\varrho }_{i}-\varrho |}{\varrho },\quad \quad \quad \text {MSE}(\hat{\varrho })=\frac{1}{\mathbb {M}}\sum _{i=1}^{\mathbb {M}}(\hat{\varrho _{i}}-\varrho )^{2}, \end{aligned}$$
  where $\hat{\varrho }$ is an estimate of $\varrho$.
10. Use Step 9 to calculate the average of estimates of $(\alpha ,\beta , \gamma )$ in addition to the mean of the estimates of RF, HRF, and MRL function with their MSEs and RABs.
11. The average of the RABs (ARAB1 and ARAB2) and average of the MSEs (AMSE1 and AMSE2) are also calculated.
12. The NACIs and LTCIs, with confidence coefficient 95%, of the parameters $(\alpha ,\beta , \gamma )$ are calculated. The average interval lengths (AILs) in addition to the coverage probabilities (COVPs) of the NACIs and LTCIs are also calculated. Further, the average of the AILs (AAIL) is calculated.

Tables 5, 6, 7, 8 and 9 present the calculations mentioned above, considering the following parameter values as exact values: $\alpha =1.5$, $\beta =0.5$, and $\gamma =0.9$. Also, the exact values of the RF, HRF, and MRL (at time $t=5.0$) are $R(5.0)=0.164667$, $h(5.0)=0.332612$, and $m(5.0)=4.72662$. Some other values have been considered through the simulation study such as: $n=25, 50, 100, 200, 400$, and $\eta =2.0$.

## Simulation results

The findings of the computations shown in Tables 5, 6, 7, 8 and 9 can be summarised as follows:

1. The results assure that the MLEs of the parameters, RF, HRF, and MRL function (at time $t=5.0$) are better than the PEs, MPSEs, and LSEs through fewer values of the ARAB1s, ARAB2s, AMSE1s, and AMSE2s.
2. Through the ARAB1s, ARAB2s, AMSE1s, and AMSE2s, the PEs of the parameters, RF, HRF, and MRL function (at time $t=5.0$) are superior to the MPSEs and LSEs.
3. Due to lower AAIL values, NACIs perform better than LTCIs.
4. The optimal value $\eta ^{*}_{D}$ is less than $\eta ^{*}_{A}$ and for higher values of *n* the optimal values $\eta ^{*}_{D}$ and $\eta ^{*}_{A}$ are each time near one to each other.
5. By increasing *n*, the RABs, MSEs, ARAB1s, ARAB2s, AMSE1s, AMSE2s, AIL, and AAIL decrease.
6. By raising the sample size *n*, the COVPs approach $95\%$.
7. The optimal values $\eta ^{*}_{D}$ and $\eta ^{*}_{A}$ decrease for higher values of the sample size *n*.

Except in rare situations, which may be consequent to fluctuations in the data, the above findings are accurate.

**Table 5:** MLEs of $(\alpha ,\beta , \gamma )$, RF, HRF, as well as the MRL with their MSEs, RABs, ARAB1s, ARAB2s, AMSE1s, and AMSE2s based on 5000 simulations.

*n*	MLE							
	$\overline{\hat{\alpha }}$	$\overline{\hat{\beta }}$	$\overline{\hat{\gamma }}$		$\overline{\hat{S}}(5.0)$	$\overline{\hat{h}}(5.0)$	$\overline{\hat{m}}(5.0)$	
	MSE	MSE	MSE	AMSE1	MSE	MSE	MSE	AMSE2
	RAB	RAB	RAB	ARAB1	RAB	RAB	RAB	ARAB2
25	1.790540	0.695866	0.949008	0.353573	0.164576	0.348134	5.327570	2.753920
	0.524506	0.222059	0.314155	0.481853	0.003566	0.008436	8.249750	0.291268
	0.360158	0.672950	0.412451		0.291269	0.203282	0.379252	
50	1.643050	0.588229	1.131130	0.342574	0.162517	0.350437	4.806850	1.139640
	0.337344	0.158547	0.531830	0.479666	0.001886	0.005551	3.411480	0.219398
	0.299532	0.597138	0.542328		0.210749	0.164875	0.282570	
100	1.569370	0.545557	1.098190	0.233691	0.163944	0.342921	4.719450	0.581844
	0.214025	0.098825	0.388224	0.396293	0.000942	0.002569	1.742020	0.160706
	0.242528	0.485147	0.461203		0.150027	0.116866	0.215224	
200	1.548070	0.530839	1.017600	0.133489	0.163978	0.339171	4.700070	0.308909
	0.131794	0.060539	0.208134	0.300917	0.000470	0.001304	0.924952	0.116607
	0.188430	0.378914	0.335406		0.104731	0.083868	0.161222	
400	1.52745	0.520045	0.954779	0.061841	0.164356	0.335514	4.723710	0.157013
	0.072573	0.03176	0.081190	0.215357	0.000235	0.000603	0.470200	0.082922
	0.142300	0.27706	0.226710		0.074760	0.058308	0.115699

**Table 6:** MPSEs of $(\alpha ,\beta , \gamma )$, RF, HRF, as well as the MRL with their MSEs, RABs, ARAB1s, ARAB2s, AMSE1s, and AMSE2s based on 5, 000 simulations.

*n*	MPSE							
	$\overline{\check{\alpha }}$	$\overline{\check{\beta }}$	$\overline{\check{\gamma }}$		$\overline{\check{S}}(5.0)$	$\overline{\check{h}}(5.0)$	$\overline{\check{m}}(5.0)$	
	MSE	MSE	MSE	AMSE1	MSE	MSE	MSE	AMSE2
	RAB	RAB	RAB	ARAB1	RAB	RAB	RAB	ARAB2
25	2.127030	1.33148	1.113470	2.622260	0.181458	0.318096	7.343700	14.84410
	3.316390	3.55651	0.993886	1.203170	0.003956	0.009371	44.51910	0.424779
	0.713731	2.08773	0.808064		0.298322	0.224143	0.751871	
50	1.839620	0.917546	1.032040	1.071230	0.174973	0.319847	6.046210	3.537090
	1.587180	1.008230	0.618292	0.757353	0.001923	0.004559	10.60480	0.269384
	0.476692	1.187960	0.607407		0.210491	0.159464	0.438198	
100	1.665420	0.702810	0.967936	0.403492	0.171428	0.322833	5.430830	1.065700
	0.578536	0.295912	0.336028	0.477487	0.000963	0.002278	3.193860	0.179351
	0.304414	0.692774	0.435273		0.150556	0.113876	0.273623	
200	1.584070	0.605248	0.926169	0.138164	0.168599	0.326338	5.114100	0.428429
	0.166746	0.091496	0.156250	0.318144	0.000478	0.001157	1.283650	0.123720
	0.205967	0.442034	0.306431		0.105974	0.081905	0.183280	
400	1.55454	0.563454	0.899518	0.062778	0.166838	0.328522	4.95441	0.18978
	0.080836	0.040175	0.067323	0.221733	0.000238	0.000569	0.568534	0.085798
	0.147728	0.30362	0.213852		0.075247	0.057668	0.12448

**Table 7:** PEs of $(\alpha ,\beta , \gamma )$, RF, HRF, as well as the MRL with their MSEs, RABs, ARAB1s, ARAB2s, AMSE1s, and AMSE2s based on 5000 simulations.

*n*	PE							
	$\overline{\ddot{\alpha }}$	$\overline{\ddot{\beta }}$	$\overline{\ddot{\gamma }}$		$\overline{\ddot{S}}(5.0)$	$\overline{\ddot{h}}(5.0)$	$\overline{\ddot{m}}(5.0)$	
	MSE	MSE	MSE	AMSE1	MSE	MSE	MSE	AMSE2
	RAB	RAB	RAB	ARAB1	RAB	RAB	RAB	ARAB2
25	2.327630	1.05515	0.949654	1.899370	0.159344	0.366258	5.720490	7.791650
	3.438310	1.85423	0.405580	0.930716	0.003790	0.021221	23.34990	0.363455
	0.757295	1.46703	0.567827		0.300378	0.268780	0.521206	
50	1.859550	0.761881	0.993496	0.645194	0.161709	0.349897	5.217670	2.881190
	1.046530	0.586547	0.302501	0.603263	0.002128	0.008768	8.632680	0.255977
	0.436760	0.888600	0.484430		0.222485	0.187616	0.357828	
100	1.661930	0.617290	0.993305	0.258641	0.163543	0.341733	4.930160	0.862772
	0.373033	0.185692	0.217199	0.416338	0.001058	0.003285	2.583970	0.177662
	0.281620	0.571220	0.396170		0.156075	0.130441	0.246470	
200	1.575280	0.555754	0.972828	0.125928	0.163817	0.337797	4.803830	0.388265
	0.160691	0.076070	0.141022	0.301632	0.000505	0.001532	1.162760	0.124986
	0.199070	0.396510	0.309320		0.108661	0.091287	0.175010	
400	1.53573	0.528377	0.947287	0.064613	0.164311	0.335342	4.76338	0.200328
	0.076804	0.035844	0.081191	0.220177	0.000259	0.000764	0.599961	0.089914
	0.14114	0.28868	0.23072		0.077461	0.064497	0.127783

**Table 8:** LSEs of $(\alpha ,\beta , \gamma )$, RF, HRF, as well as the MRL with their MSEs, RABs, ARAB1s, ARAB2s, AMSE1s, and AMSE2s based on 5000 simulations.

*n*	LSE							
	$\overline{\bar{\alpha }}$	$\overline{\bar{\beta }}$	$\overline{\bar{\gamma }}$		$\overline{\bar{S}}(5.0)$	$\overline{\bar{h}}(5.0)$	$\overline{\bar{m}}(5.0)$	
	MSE	MSE	MSE	AMSE1	MSE	MSE	MSE	AMSE2
	RAB	RAB	RAB	ARAB1	RAB	RAB	RAB	ARAB2
25	2.135020	0.884413	1.020580	1.513970	0.165975	0.361049	6.163780	9.713760
	3.085950	0.997892	0.458082	0.809172	0.003723	0.032027	29.10550	0.397638
	0.652100	1.201070	0.574350		0.295060	0.291868	0.605986	
50	1.78580	0.730437	1.030610	0.694684	0.167815	0.340113	5.564220	3.038030
	1.25444	0.457900	0.371714	0.594907	0.002049	0.007841	9.104190	0.274031
	0.41262	0.858740	0.513360		0.219713	0.192503	0.409877	
100	1.631430	0.643337	0.998255	0.272049	0.167523	0.334144	5.211260	1.291900
	0.334998	0.224562	0.256586	0.441315	0.001113	0.003447	3.871140	0.192611
	0.278240	0.626020	0.419690		0.160967	0.133156	0.283712	
200	1.567070	0.574814	0.977946	0.140616	0.165754	0.334351	4.952960	0.551822
	0.155125	0.094997	0.171727	0.326558	0.000572	0.001795	1.653100	0.138986
	0.199760	0.444630	0.335290		0.115557	0.098500	0.202901	
400	1.53394	0.537726	0.941905	0.066317	0.165766	0.332979	4.85058	0.261504
	0.073841	0.040786	0.084326	0.230508	0.000293	0.000874	0.783343	0.098183
	0.14293	0.30921	0.23938		0.082918	0.068978	0.142651

**Table 9:** AILs and COVPs (in %) of 95% CIs of $\alpha , \beta$ and $\gamma$ in additional to the optimal value of $\eta$ based on 5000 simulations.

*n*	NACI			LTCI			Optimal value	
	COVP$(\alpha )$	AIL$(\alpha )$		COVP$(\alpha )$	AIL$(\alpha )$			
	COVP$(\beta )$	AIL$(\beta )$		COVP$(\beta )$	AIL$(\beta )$			
	COVP$(\gamma )$	AIL$(\gamma )$	AAIL	COVP$(\gamma )$	AIL$(\gamma )$	AAIL	$\eta ^{*}_{D}$	$\eta ^{*}_{A}$
25	95.88	4.00846	2.95886	98.36	5.80898	6.11456	1.57213	2.39464
	93.04	2.24527		98.92	6.83400			
	99.24	2.62286		99.90	5.70072			
50	93.46	3.01404	2.48743	97.70	3.54504	4.05261	1.64043	2.11457
	87.84	1.62601		97.68	3.55237			
	97.34	2.82225		98.92	5.06041			
100	93.28	2.13763	1.91559	96.14	2.31503	2.54792	1.46143	1.67466
	90.40	1.25834		97.12	1.92872			
	94.22	2.35079		97.94	3.40000			
200	96.58	1.51371	1.38399	95.30	1.57596	1.56073	1.34732	1.29226
	92.46	0.98087		96.20	1.16576			
	93.10	1.65740		95.68	1.94047			
400	95.58	1.05793	0.87534	95.84	1.0794	0.91335	1.24956	0.97445
	93.72	0.69274		96.04	0.7473			
	93.34	1.05039		95.6	1.11072			

## Concluding remarks

Under step-stress PALTs, we have estimated the distribution parameters of the TMR system, with each component’s lifetime following a LD. Additionally, we have calculated the RF, HRF, and MRL of the TMR system. Various estimation methods, including ML, percentile, LS, and MPS, have been taken into account. The determination of the optimal stress change time has been achieved using two distinct optimality criteria. An illustrative instance involving two actual datasets has been examined. Additionally, a Monte Carlo simulation has been utilized to analyze the effectiveness of the estimation methodologies. The assessment of these estimation techniques has been executed, uncovering that the ML method outperforms the remaining three methods in accuracy and performance as indicated by the numerical results.

### Future research

Neutrosophic statistics is an extension of classical statistics that deals with uncertain, indeterminate, and inconsistent data. It is based on the concept of neutrosophy. It offers a new framework for modeling and analyzing data coming from complex process or uncertain environment. It has been applied in various fields, such as decision-making, risk assessment, medical diagnosis, and pattern recognition, where uncertainties play a significant role. Therefore, the current study can be extended using neutrosophic statistics as future research, see, for example,^39,40^.

## Data Availability

All data generated or analysed during this study are included in this article.
